# Advantages and Disadvantages of Commonly Used Urinary Incontinence Questionnaires—How to Correctly Choose Questionnaire in Urinary Incontinence Diagnosis?

**DOI:** 10.3390/jcm14228196

**Published:** 2025-11-19

**Authors:** Katarzyna Skorupska, Aleksandra Kamińska

**Affiliations:** 2nd Department of Gynecology, Medical University of Lublin, 20-090 Lublin, Poland; kasiaperzylo@hotmail.com

**Keywords:** urinary incontinence, quality of life, questionnaires

## Abstract

**Background/Objectives:** The aim of this study was to describe the most clinically useful questionnaires in UI diagnosis and determine their optimal applicability to specific types of UI. **Methods:** The MEDLINE (PubMed), EMBASE, and Scopus databases were searched using the following MeSH terms in titles and abstracts: (“questionnaires and urinary incontinence” OR “questionnaires and quality of life”). No language restrictions were applied, and the search was limited to studies involving human subjects. **Results:** Numerous methods are available to assess the severity of incontinence and its symptoms. Objective measures recommended for the evaluation of urinary incontinence in women include simple investigations combined with thorough clinical history taking. However, these methods alone do not adequately capture patients’ perceptions of their symptoms or their impact on quality of life. To obtain a comprehensive assessment, the International Consultation on Incontinence recommends combining subjective and objective measures. Thus, evaluating urinary incontinence should include both the severity of symptoms and the extent to which they affect daily life. **Conclusions:** Questionnaires (Qs), as patient-reported outcome (PRO) measures, are valuable tools for identifying and diagnosing dysfunction, assessing its severity, evaluating its impact on quality of life (QoL), and measuring improvement or satisfaction following treatment. Accurate assessment of clinical symptoms and their impact on health-related QoL has become central to contemporary medical outcomes research.

## 1. Introduction

Urinary incontinence, defined as the involuntary loss of urine, is a significant and diverse global health concern. This condition includes several subtypes, such as stress urinary incontinence (SUI), overactive bladder (OAB), and mixed urinary incontinence (MUI). According to the International Continence Society (ICS) and the International Urogynecological Association (IUGA), OAB is characterized by urinary urgency, typically accompanied by increased daytime frequency and/or nocturia, with (OAB-wet) or without (OAB-dry) urinary incontinence, in the absence of urinary tract infection or any other identifiable pathology.

SUI is defined as the involuntary leakage of urine during physical effort; exertion; or activities such as exercise, sneezing, or coughing. MUI includes symptoms of both stress and urgency urinary incontinence, involving involuntary urine loss associated with urgency as well as with exertional activities like sports, sneezing, or coughing [1].

Although urinary incontinence is prevalent across populations, its clinical significance can vary; even individuals reporting severe symptoms may not always experience substantial distress [2]. Therefore, a comprehensive evaluation of its impact must consider both the severity of symptoms and the extent to which they interfere with daily functioning.

Patient-reported outcome measures (PROMs), often in the form of standardized questionnaires, are essential for identifying and diagnosing urinary dysfunction, assessing symptom burden, evaluating the impact on quality of life (QoL), and monitoring treatment outcomes. Accurate assessment of clinical symptoms and their influence on health-related QoL has become a central aspect of outcomes research in medicine. The use of such questionnaires has been increasingly adopted in the field of urogynecology. Conventional urodynamic studies and existing diagnostics have limitations in addressing the heterogeneity of SUI. Some authors emphasise that a more patient-centred, personalised diagnostic pathway is necessary to enhance decision-making in clinical practice. While questionnaires can capture the symptom burden, psychological impact, and sexual/quality-of-life domains, they are not sufficient by themselves as a full diagnostic tool. They need to be integrated with imaging, physical examination and other diagnostic methods [3]

The aim of this study was to present the most valuable questionnaires used in the diagnosis of urinary incontinence (UI) and to indicate which of them are appropriate for specific UI types.

## 2. Materials and Methods

**Figure 1:**
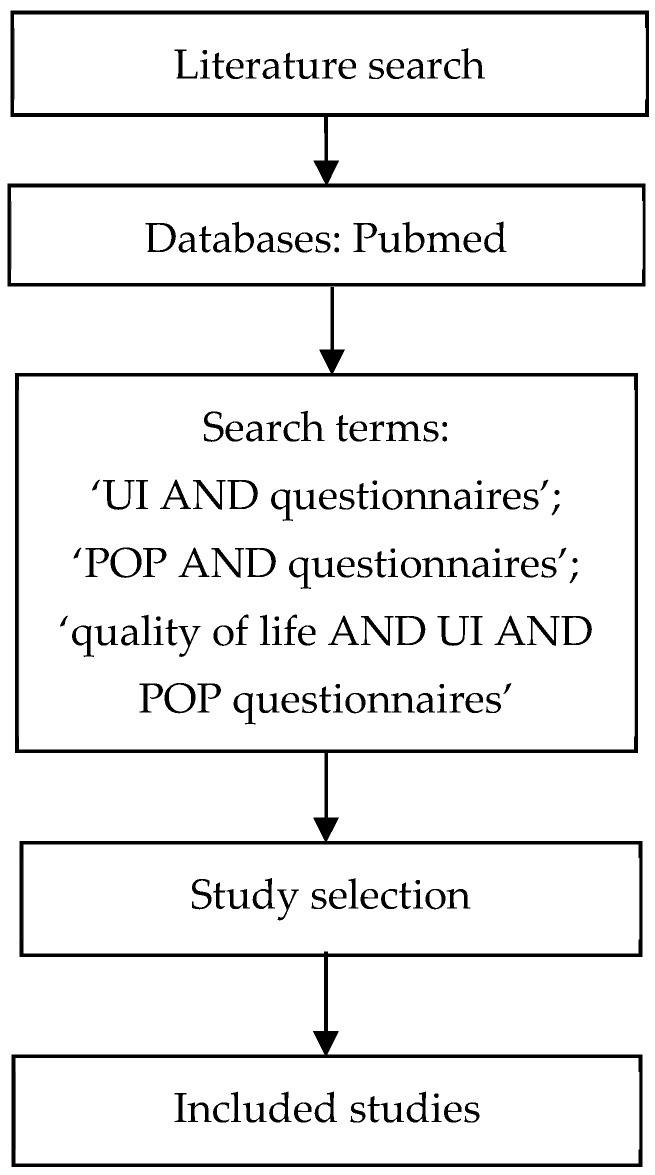
A conceptual overview figure.

Below is a description of the most commonly used questionnaires in urogynecology to assess quality of life and the severity of symptoms associated with urinary incontinence. Study selection process is presented in Figure 1.

### 2.1. The International Consultation on Incontinence Modular Questionnaire (ICIQ)—Grade: A

The International Consultation on Incontinence Questionnaire (ICIQ) consists of 19 validated modules designed to assess a wide range of pelvic floor and lower urinary tract symptoms. The core modules include assessments of urinary symptoms in men (ICIQ-MLUTS) and women (ICIQ-FLUTS), vaginal symptoms (ICIQ-VS), bowel symptoms (ICIQ-B), and urinary incontinence (ICIQ-UI Short Form).

In addition to these core tools, the ICIQ includes modules tailored to specific patient populations and conditions. These include nocturia (ICIQ-N), overactive bladder (ICIQ-OAB), underactive bladder (ICIQ-UAB), neurogenic bowel dysfunction (ICIQ-NeuroBowel), pediatric populations (ICIQ-CLUTS), inflammatory bowel disease (ICIQ-IBD), and cognitively impaired older adults (ICIQ-Cog).

Furthermore, quality of life (QoL) modules have been developed for individuals using long-term urinary catheters (ICIQ-LTCQoL) and absorbent products (ICIQ-PadPROM) [4].

### 2.2. International Consultation on Incontinence Questionnaire—Urinary Incontinence Module (ICIQ-UI Short Form)—Grade: A

The International Consultation on Incontinence Questionnaire—Urinary Incontinence Short Form (ICIQ-UI SF) is a widely used tool for assessing the frequency, severity, and impact of urinary incontinence on quality of life in both men and women. Suitable for use in both research and clinical practice, the ICIQ-UI SF generates a total score ranging from 0 to 21, with higher scores indicating greater symptom severity.

It is routinely used by general practitioners and specialists in both primary and secondary care settings to screen for urinary incontinence, summarize perceived causes and impacts of symptoms, and facilitate structured communication between clinicians and patients.

The questionnaire consists of four items: frequency of urinary incontinence, amount of leakage, overall impact of incontinence on daily life, and a self-diagnostic item that allows patients to identify perceived causes of their symptoms [5].

The Third International Consultation on Incontinence recommended the use of validated instruments such as the ICIQ in all randomized controlled trials assessing the efficacy of treatments for urinary incontinence, in order to measure their effects on patient-centered outcomes [6]. The ICIQ-UI SF is sensitive to clinically meaningful changes following intervention and can distinguish between varying degrees of improvement resulting from different therapeutic approaches.

Research has shown that among patients with urodynamically confirmed stress urinary incontinence (SUI), higher ICIQ-UI SF scores are associated with greater symptom severity [7]. Additionally, studies comparing questionnaire data with urodynamic findings have highlighted the tool’s diagnostic utility in identifying mixed urinary incontinence (MUI), although no significant correlation was observed in the SUI group [8]. Nyström et al. in their study analysed the minimal important difference (MID) of the ICIQ--UI SF (International Consultation on Incontinence Questionnaire—Urinary Incontinence Short Form) after self-management for urinary incontinence. It provides evidence of how much change on the questionnaire score reflects a meaningful improvement from the patient’s perspective [9].

The ICIQ is considered a valuable instrument for outcome measurement in clinical trials, epidemiological studies, and routine clinical evaluation. It is widely regarded as the gold standard for identifying urinary incontinence and has been translated into 43 languages [10]. Furthermore, the ICIQ-UI SF has shown a strong correlation with objective measures such as the 24 h pad test, and its total score has also been found to correlate well with urodynamic parameters [11].

### 2.3. International Consultation on Incontinence Questionnaire—Overactive Bladder Module (ICIQ-OAB)—Grade: A

The International Consultation on Incontinence Questionnaire—Overactive Bladder (ICIQ-OAB) is a validated tool designed to assess symptoms of overactive bladder (OAB), their impact on quality of life (QoL), and treatment outcomes in both men and women, across clinical and research settings. The questionnaire consists of four items addressing the core symptoms of OAB: urinary frequency, nocturia, urgency, and urgency urinary incontinence (UUI). The total score ranges from 0 to 16, with higher scores indicating greater symptom severity [4].

The International Consultation on Incontinence recommends that the ICIQ-OAB be used alongside the ICIQ-UI Short Form to provide a more comprehensive assessment of lower urinary tract symptoms. The ICIQ-OAB has also been used as both a diagnostic and evaluative instrument in studies comparing its performance with urodynamic assessments in patients undergoing treatment for OAB [12].

Evidence suggests that patients diagnosed with OAB via urodynamic evaluation experienced greater symptom improvement following treatment than those without urodynamic confirmation. Furthermore, slightly greater reductions in symptom severity were reported among individuals who underwent urodynamic assessment and met the ICIQ-OAB criteria, with over half of the women in these studies reporting long-term symptom improvement [12].

### 2.4. King’s Health Questionnaire (KHQ)—Grade: A

The King’s Health Questionnaire (KHQ) is a condition-specific, validated instrument designed to assess health-related quality of life (HRQoL) in individuals affected by urinary incontinence. The KHQ comprises 21 items divided into two main parts: Part I evaluates general health (GH) and the impact of incontinence, while Part II includes multiple subdomains such as role limitations—emotional (RE) and physical (RP), social limitations (SL), personal relationships (PR), emotions (E), sleep/energy (S/E), and symptom severity (SM). Higher KHQ scores indicate greater impairment in quality of life [13].

KHQ scores are converted to a scale ranging from 0 to 100, where 0 denotes no impairment and 100 represents the maximum level of QoL impairment. As the KHQ uses a categorical scoring system, non-parametric statistical analyses are recommended, with central tendency best represented by medians rather than means. A change of at least 5 points in most domains (except for GH and SM, where a 3–4 point change is considered clinically significant) reflects a meaningful improvement in HRQoL following treatment. For a medium effect size, typical domain changes range from 12 to 15 points, or 6 to 11 points for GH and SM [14].

The reliability and validity of the KHQ have been confirmed in patients with overactive bladder (OAB) [15]. In clinical trials, the KHQ is commonly used to evaluate patient-perceived treatment benefits. A minimally important difference (MID) has been defined as an improvement of >5% from baseline in each domain, which is particularly useful in comparisons of active treatments (e.g., solifenacin) with placebo [16]. Moreover, the KHQ has proven effective in evaluating postoperative outcomes in women undergoing surgery for stress urinary incontinence (SUI), with a clinically meaningful threshold established at ≥10 points [17].

However, evidence suggests that the KHQ has certain limitations in distinguishing between specific types of urinary incontinence. In comparative studies involving questionnaire results and urodynamic findings, the KHQ showed a negative correlation with both mixed urinary incontinence (MUI) and SUI, suggesting limited diagnostic specificity [8]. These findings are supported by further research indicating that the KHQ is more effective for assessing overall health and well-being rather than for differentiating between subtypes of urinary incontinence [18].

Nevertheless, KHQ domain scores have shown significant associations with the severity of urodynamic stress incontinence (USI). Grigoriadis et al. reported that all domains—except general health perception and personal relationships—had significantly higher mean values in patients with more severe USI, as graded by the “1-3-5 cough test” conducted during cystometric filling. This test categorizes severity based on the number of coughs required to provoke leakage: mild (leakage after five coughs), moderate (after three), or severe (after one), providing an objective method for interpreting SUI severity [19].

In addition, the KHQ has been used to assess symptom severity in women with MUI. In one study, patient responses to the KHQ helped identify the most bothersome subtype of incontinence (stress or urge) and determine the predominant symptom pattern. These responses were compared with urodynamic diagnoses, revealing that women with urge-predominant MUI were more likely to exhibit detrusor overactivity, whereas those with stress-predominant MUI more often presented with urodynamic stress incontinence. These findings support the KHQ’s utility in symptom profiling and in distinguishing MUI subtypes [20].

To date, the KHQ has been adapted into eight different formats and translated into 45 languages, further demonstrating its global applicability and relevance [16].

### 2.5. The Urogenital Distress Inventory Short Form (UDI-6)—Grade: A

The Urogenital Distress Inventory–Short Form (UDI-6) is a validated, concise six-item questionnaire developed to evaluate symptoms related to lower urinary tract dysfunction and pelvic organ prolapse. Each item is rated on a 4-point Likert scale ranging from 0 (“not at all”) to 3 (“greatly”). The overall score is derived by calculating the mean of the item scores and multiplying by 33.3, thus transforming the results onto a 0 to 100 scale. Elevated scores correspond to increased symptom burden and associated impairment [21].

This brief instrument is a shortened version of the original 19-item UDI, a condition-specific quality of life measure [22]. The UDI-6 covers six key symptoms:

1. Frequent urination;
2. Leakage triggered by urgency;
3. Leakage linked to physical exertion;
4. Leakage occurring during coughing or sneezing (small volumes);
5. Difficulty with bladder emptying;
6. Discomfort or pain in the lower abdomen or genital region.

These items are categorized into three clinically relevant domains:

- Irritative Symptoms (IS): items 1 and 2;
- Stress Symptoms (SS): items 3 and 4;
- Obstructive/Discomfort Symptoms (OS): items 5 and 6 [23].

One of the primary advantages of the UDI-6 lies in its brevity and organized symptom classification, allowing for rapid yet comprehensive screening across the range of urogenital complaints. Its streamlined format has led to widespread adoption in clinical settings, often replacing the more extensive original UDI.

Numerous investigations have confirmed its psychometric properties and clinical relevance. For instance, a study conducted in Arabic-speaking populations revealed a significant association between UDI-6 scores and urodynamic results in women diagnosed with stress urinary incontinence (SUI) [24]. Similarly, research from Poland demonstrated strong reliability for the UDI-6 among patients with SUI and overactive bladder (OAB), though its performance was less robust in women with mixed urinary incontinence (MUI), implying limited diagnostic accuracy in this subgroup [25].

Lemack and colleagues further observed that particular UDI-6 item responses could serve as predictors for urodynamic diagnoses including SUI, detrusor instability (DI), and bladder outlet obstruction. These findings suggest that the UDI-6 may aid clinicians in identifying patients who would benefit from comprehensive urodynamic evaluation [26].

While some researchers have utilized the UDI-6 to discern the predominant subjective subtype of urinary incontinence, others have expressed reservations about its capacity to accurately classify MUI [27,28]. Brubaker et al. employed the full 19-item UDI to categorize MUI phenotypes. By analyzing responses related to urgency and exertion-induced leakage, they stratified patients into stress-predominant, urge-predominant, and mixed symptom groups using the ratio of the Irritative (UDI-i) and Stress (UDI-s) subscale scores. However, receiver operating characteristic (ROC) curve analysis did not establish definitive cutoff values, restricting the tool’s utility for rigorous MUI classification or prognostication [29].

Despite certain drawbacks, especially regarding its diagnostic specificity for MUI, the UDI-6 remains a practical, efficient, and extensively utilized tool. Its primary strength is the clear and clinically meaningful division of symptoms into three domains, offering a broad yet concise overview of urogenital distress, while maintaining ease of use and interpretability [21].

### 2.6. The Incontinence Impact Questionnaire Short Form (IIQ-7)—Grade: A

The Incontinence Impact Questionnaire–Short Form (IIQ-7) is a validated, condition-specific tool designed to assess the impact of urinary incontinence (UI) on various aspects of daily living, social functioning, and emotional well-being. The IIQ-7 measures how UI disrupts daily activities, interpersonal relationships, and psychological health. Psychometric analyses based on classical test theory have confirmed its strong internal consistency, good test–retest reliability, and a unidimensional factor structure [30].

This questionnaire consists of seven items evaluating the extent to which UI affects:

1. Household chores;
2. Physical recreation;
3. Entertainment activities;
4. Travel beyond 30 min from home;
5. Social interactions;
6. Emotional well-being;
7. Feelings of frustration.

These items are grouped into four conceptual domains:

- Physical Activity (PA): items 1 and 2;
- Travel (TR): items 3 and 4;
- Social Activities (SA): item 5;
- Emotional Health (EH): items 6 and 7 [23].

The IIQ-7 is widely used in both research and clinical settings to evaluate the psychosocial burden of UI in women, including those undergoing treatment for stress urinary incontinence (SUI) [31] and mixed urinary incontinence (MUI) [16]. Its sensitivity to changes in quality of life (QoL) before and after treatment has made it a valuable outcome measure across various incontinence subtypes.

A Polish study reported excellent internal consistency of the IIQ-7 among patients with SUI, overactive bladder (OAB), and MUI, supporting its applicability across diverse clinical populations [25]. Corcos et al. employed a neural network (NN) model to establish clinically relevant cut-off values for the IIQ-7:

- Scores below 50 are associated with good QoL;
- Scores between 50 and 70 indicate moderate QoL;
- Scores above 70 reflect poor QoL [32].

The authors proposed that these thresholds might guide clinical decisions—for example, patients scoring below 50 may not experience significant benefit from surgical intervention, whereas those with scores above 70 could expect more pronounced postoperative improvement.

However, the relationship between objective urodynamic parameters and IIQ-7 scores remains unclear. Arribillaga et al. found no significant correlation between abdominal leak point pressure during urodynamic testing and symptom severity or QoL impact as measured by the IIQ-7 in women with SUI [33]. This suggests that while the IIQ-7 is valuable for capturing subjective symptom burden and treatment response, it may not reliably reflect underlying urodynamic abnormalities.

In summary, the IIQ-7 is a concise and psychometrically sound instrument well-suited for assessing the psychosocial effects of urinary incontinence and for monitoring treatment outcomes across a variety of patient populations.

### 2.7. Overactive Bladder Questionnaire (OAB-q)—Grade: A

The original Overactive Bladder Questionnaire (OAB-q) included 62 items, comprising 13 questions focused on symptoms and 44 items addressing health-related quality of life (HRQoL). Its shortened form, widely implemented in both clinical settings and research, features an eight-item symptom bother scale plus 25 questions spanning four HRQoL domains: coping strategies, concerns, sleep disturbances, and social engagement.

What sets the OAB-q apart is that it was developed based on direct input from both continent and incontinent patients experiencing overactive bladder (OAB), which ensures the questionnaire remains closely aligned with patient experiences. Psychometric testing has confirmed its high reliability, validity, and sensitivity, particularly in capturing changes in hallmark OAB symptoms like urgency, increased frequency, and nocturia.

The questionnaire effectively distinguishes patients with different levels of nocturia severity, as well as those with stress, urge, or mixed urinary incontinence, especially in community-based samples. The significant disparities observed in scores between OAB sufferers and control subjects underscore its sensitivity and clinical relevance.

The OAB-q’s responsiveness to antimuscarinic medications is well established, with notable improvements seen across all subscales at 4 and 12 weeks post-treatment. These improvements align closely with reductions in urgency episodes, frequency of urination, and clinical evaluations of patient outcomes [34,35,36].

Nonetheless, additional studies involving larger, more diverse populations are necessary to further validate and confirm the OAB-q’s utility as a reliable instrument for assessing patients with urinary incontinence and lower urinary tract symptoms (LUTS).

### 2.8. ICIQ Incontinence Quality of Life Questionnaire (I-QOL): Instrument Description and Clinical Application)—Grade: A

The ICIQ Incontinence Quality of Life Questionnaire (I-QOL) is a 22-item self-reported tool specifically created for clinical research to measure the impact of urinary incontinence on patients’ quality of life. While originally developed for use in both men and women, the majority of psychometric validation has been conducted predominantly in female cohorts.

Extensive validation studies have demonstrated the I-QOL’s excellent reliability and validity, particularly highlighting the robustness of the total score over individual subscale scores. The questionnaire is divided into three distinct subdomains, with consistent evidence supporting the combined interpretation of these subscales alongside the overall summary score across multiple culturally adapted versions worldwide.

Among diverse patient populations, I-QOL scores have shown strong associations with the perceived severity of urinary incontinence, patterns of healthcare utilization, and episode frequency, underscoring the tool’s sensitivity to clinical symptom burden. It is widely implemented as a primary outcome measure in randomized controlled trials evaluating treatments for stress urinary incontinence (SUI) and has been instrumental in validating patient-reported global impression measures [37,38,39,40].

## 3. Discussion

There are numerous methods available to quantify the severity of urinary incontinence and the symptoms experienced by patients. Objective evaluation in women typically involves straightforward procedures such as urine dipstick testing and pelvic floor examination, combined with a detailed clinical history. However, these objective measures alone do not fully capture the patient’s subjective experience or the impact of symptoms on quality of life (QoL). To address this limitation, the International Consultation on Incontinence (ICI) recommends using both objective assessments and patient-reported outcome measures (PROMs) to achieve a more comprehensive evaluation [41].

PROMs have become essential endpoints in clinical trials that assess the effectiveness of incontinence treatments. For these instruments to be clinically valuable, they must demonstrate sufficient validity, reliability, and responsiveness. Responsiveness refers to the tool’s ability to detect clinically meaningful changes following an intervention. However, a measure can be responsive without necessarily reflecting the smallest change that patients consider important.

This has led to increased focus on the concept of the Minimum Clinically Important Difference (MCID), defined as the smallest change in an outcome score that patients perceive as beneficial and meaningful. Understanding the MCID has several advantages: (1) it guides researchers in designing studies and reporting the proportion of treatment responders; (2) it helps clinicians interpret results that are clinically relevant beyond mere statistical significance; and (3) it facilitates clearer communication with patients regarding expected treatment outcomes [42].

According to Bowling, the evaluation of questionnaires should emphasize their psychometric properties—particularly crucial when measuring complex, subjective constructs such as QoL or perceived health status, which cannot be directly assessed through simple tests [43]. The two fundamental psychometric properties are reliability and validity. Reliability refers to the consistency of a measurement tool, reflecting its ability to produce stable and reproducible results over repeated administrations.

Validity testing often includes assessing criterion validity, which examines how well a questionnaire correlates with a more objective or “gold standard” measure of the same domain. Face validity is also important to ensure the instrument’s relevance and practical applicability in clinical settings.

Responsiveness, distinct from reliability and validity, measures how well a questionnaire can detect meaningful changes over time within a patient population. For example, improvements in continence symptoms should align with corresponding improvements in questionnaire scores.

Lastly, the practical aspects of questionnaire use must be taken into account, especially in busy clinical environments. Ease of use and the simplicity of scoring systems are key factors influencing the acceptance and routine implementation of PROMs by healthcare professionals [41,43].

It is important to highlight less commonly used questionnaires that are primarily utilized in scientific research.

Among these, the Symptom Impact Index for Stress Incontinence in women consists of three items that evaluate the extent to which women avoid certain activities due to stress urinary incontinence (SUI). While this instrument has demonstrated good reliability, its validity was found to correlate strongly with body mass index and showed no significant association with whether the patient had undergone surgical treatment for SUI [44,45].

The Urinary Incontinence Severity Score includes ten items, with four assessing the volume of leakage and six addressing the impact of urinary incontinence on daily living. A limitation of this tool is that it does not separately measure symptom impact or quality of life, as it provides only a combined total score reflecting both severity and impact [46].

The Urge Impact Scale is a 24-item questionnaire designed to assess the quality-of-life effects of urge urinary incontinence in older adults. Although only one validation study with a small sample size (48 participants, with 27 completing the final questionnaire) has been published, it reported favorable psychometric properties [47].

The Urinary Incontinence Handicap Inventory comprises 17 items that evaluate the emotional, social, and physical impact of urinary incontinence, specifically targeting elderly women with detrusor instability. This instrument has demonstrated strong internal consistency, excellent test–retest reliability, and solid construct validity [48].

The York Incontinence Perception Scale is an eight-item tool developed to assess the psychological impact of urinary incontinence. Validation studies have shown that it possesses adequate internal consistency, construct validity, and responsiveness to treatment-related changes [49].

Among the questionnaires reviewed, the Incontinence Impact Questionnaire (IIQ), King’s Health Questionnaire (KHQ), and Urogenital Distress Inventory (UDI) demonstrated the strongest psychometric properties. When considering additional factors such as ease of use and clinical applicability, the KHQ, Incontinence Quality of Life Questionnaire (I-QOL), and International Consultation on Incontinence Questionnaire–Short Form (ICIQ-SF) are recommended for routine clinical practice [41].

To facilitate clinical comparison, Table 1 summarizes the principal characteristics, psychometric strengths, and intended applications of the most commonly used questionnaires in urogynecology. This overview supports the selection of appropriate instruments for clinical and research purposes.

## 4. Conclusions

Among the urinary incontinence-specific instruments, the King’s Health Questionnaire (KHQ) stands out as the most extensively validated tool, having been tested across the largest and most diverse patient populations, and is available in numerous language versions. Other instruments with strong psychometric properties, such as the Incontinence Impact Questionnaire (IIQ) and the Incontinence Quality of Life Questionnaire (I-QOL), are also highly recommended for use in female patients. When reducing the burden on respondents is a key consideration, the shorter IIQ-7 version serves as an efficient and reliable alternative.

For evaluating health-related quality of life (HRQoL) in patients with overactive bladder (OAB), the OAB-q is unique in that it was specifically developed and validated for both continent and incontinent individuals with OAB. As such, the OAB-q is likely the most suitable instrument for diverse patient populations exhibiting symptoms such as urgency, frequency, or nocturia, regardless of the presence of incontinence [34].

Finally, we would like to present practical recommendations enabling the use of questionnaires in everyday medical practice.

Routine Clinical Practice
  - For initial screening and severity assessment, the ICIQ-UI Short Form or UDI-6 are recommended.
    → Both are brief, easy to administer, and offer rapid insight into symptom burden and impact on daily functioning.
  - The KHQ and I-QOL are valuable for comprehensive quality-of-life assessment in routine follow-up and post-treatment evaluation.
  - Combining a symptom questionnaire (e.g., ICIQ-UI SF) with a QoL measure (e.g., KHQ or I-QOL) provides the most holistic assessment.

Research and Clinical Trials
  - Studies evaluating treatment outcomes or longitudinal changes should prioritize instruments with established responsiveness and defined MCID values, such as the KHQ, I-QOL, and OAB-q.
  - For differentiating between urinary incontinence subtypes (SUI, UUI, MUI, OAB), combined use of ICIQ-UI SF and ICIQ-OAB is advisable, as they complement each other in symptom profiling.

Target Population Considerations
  - The ICIQ Modular System provides flexibility for diverse patient groups, including men, women, pediatric populations, and cognitively impaired individuals.
  - For older adults or patients with multiple comorbidities, shorter tools such as UDI-6 and IIQ-7 minimize respondent fatigue while maintaining clinical relevance.

Diagnostic vs. Follow-Up Use
  - Screening and Diagnostic Purposes: ICIQ-UI SF, UDI-6, ICIQ-OAB.
  - Monitoring and Outcome Evaluation: KHQ, I-QOL, OAB-q, IIQ-7.

## Figures and Tables

**Table 1:** Comparative summary of validated questionnaires used in urogynecology.

Questionnaire	No. of Items	Psychometric Performance	Target Population	Clinical Purpose	Key Domains/Notes
ICIQ (Modular System)	19 modules (varies per module)	Excellent validity, reliability, and responsiveness; widely validated and translated into 43 languages	Men, women, pediatric, cognitively impaired, neurogenic, etc.	Screening, diagnosis, follow-up	Comprehensive modular approach covering urinary, vaginal, bowel, and QoL domains
ICIQ-UI Short Form	4	High internal consistency and sensitivity to change; correlates with pad test and urodynamics	Men and women with urinary incontinence	Screening, diagnosis, follow-up	Frequency, severity, and QoL impact of UI
ICIQ-OAB	4	Strong validity and responsiveness; aligns with urodynamic findings	Men and women with overactive bladder	Diagnosis, follow-up	Frequency, nocturia, urgency, UUI
King’s Health Questionnaire (KHQ)	21	Excellent reliability and validity; clinically meaningful change ≥5–10 points	Women with UI (SUI, OAB, MUI)	Diagnosis, follow-up	HRQoL across multiple domains (physical, social, emotional)
UDI-6	6	High reliability and validity; responsive to treatment; good for screening	Women with SUI, OAB, POP	Screening, follow-up	Irritative, stress, and obstructive/discomfort symptoms
IIQ-7	7	Strong psychometric properties; unidimensional; good test–retest reliability	Women with SUI, OAB, MUI	Follow-up, treatment outcome	Physical, travel, social, and emotional impact
OAB-q (short form)	33 (8 symptom + 25 HRQoL)	Excellent internal consistency and sensitivity; responsive to therapy	Men and women with OAB	Diagnosis, follow-up	Symptom bother and HRQoL (coping, concern, sleep, social)
I-QOL (ICIQ module)	22	Excellent reliability and validity; sensitive to severity and treatment response	Men and women (validated mainly in women)	Diagnosis, follow-up	Avoidance, psychosocial impact, embarrassment

## Data Availability

No new data were generated or analyzed in support of this research.

## Abbreviations

The following abbreviations are used in this manuscript:

UI	urinary incontinence
SUI	stress urinary incontinence
OAB	overactive bladder
MUI	mixed urinary incontinence
ICS	International Continence Society
IUGA	International Urogynecological Association
PROMs	Patient-reported outcome measures
QoL	quality of life
ICIQ	The International Consultation on Incontinence Questionnaire
ICIQ-UI SH	International Consultation on Incontinence Questionnaire—Urinary Incontinence Module (short form)
ICIQ-OAB	International Consultation on Incontinence Questionnaire—Overactive Bladder Module
KHQ	King’s Health Questionnaire
HRQoL	health-related quality of life
GH	general health
RE	role limitations—emotional
RP	role limitations-physical
SL	social limitations
PR	personal relationships
E	emotions
S/E	sleep/energy
SM	symptom severity
MID	minimally important difference
USI	urodynamic stress incontinence
UDI- 6	The Urogenital Distress Inventory–Short Form
IS	Irritative Symptoms
SS	Stress Symptoms
OS	Obstructive/Discomfort Symptoms
DI	detrusor instability
ROC	receiver operating characteristic
IIQ-7	The Incontinence Impact Questionnaire Short Form
PA	Physical Activity
TR	Travel
SA	Social Activities
EH	Emotional Health
NN	neural network
OAB-q	Overactive Bladder Questionnaire
LUTS	lower urinary tract symptoms
ICI	the International Consultation on Incontinence
